# GATA6 Activates Wnt Signaling in Pancreatic Cancer by Negatively Regulating the Wnt Antagonist Dickkopf-1

**DOI:** 10.1371/journal.pone.0022129

**Published:** 2011-07-19

**Authors:** Yi Zhong, Zheng Wang, Baojin Fu, Fan Pan, Shinichi Yachida, Mousumi Dhara, Emilia Albesiano, Li Li, Yoshiki Naito, Felip Vilardell, Christopher Cummings, Paola Martinelli, Ang Li, Raluca Yonescu, Qingyong Ma, Constance A. Griffin, Francisco X. Real, Christine A. Iacobuzio-Donahue

**Affiliations:** 1 Department of Pathology, The Sol Goldman Pancreatic Cancer Research Center, Johns Hopkins Medical Institutions, Baltimore, Maryland, United States of America; 2 Department of Oncology, The Sol Goldman Pancreatic Cancer Research Center, Johns Hopkins Medical Institutions, Baltimore, Maryland, United States of America; 3 Department of Surgery, The Sol Goldman Pancreatic Cancer Research Center, Johns Hopkins Medical Institutions, Baltimore, Maryland, United States of America; 4 University of Colorado School of Medicine, Denver, Colorado, United States of America; 5 Hospital Universitari Arnau de Vilanova, Lleida, Spain; 6 Programa de Patologia Molecular, Centro Nacional de Investigaciones Oncológicas, Madrid, Spain; 7 Department of Hepatobiliary Surgery, The First Affiliated Hospital, School of Medicine of Xi'an Jiaotong University, Shanxi, China; 8 Department of Opthamology, The First Affiliated Hospital, School of Medicine of Xi'an Jiaotong University, Shanxi, China; 9 Departament de Ciències Experimentals i de la Salut, Universitat Pompeu Fabra, Barcelona, Spain; The University of Hong Kong, Hong Kong

## Abstract

Pancreatic ductal adenocarcinoma (PDAC) is a highly lethal disease characterized by late diagnosis and treatment resistance. Recurrent genetic alterations in defined genes in association with perturbations of developmental cell signaling pathways have been associated with PDAC development and progression. Here, we show that GATA6 contributes to pancreatic carcinogenesis during the temporal progression of pancreatic intraepithelial neoplasia by virtue of Wnt pathway activation. *GATA6* is recurrently amplified by both quantitative-PCR and fluorescent in-situ hybridization in human pancreatic intraepithelial neoplasia and in PDAC tissues, and *GATA6* copy number is significantly correlated with overall patient survival. Forced overexpression of GATA6 in cancer cell lines enhanced cell proliferation and colony formation in soft agar *in vitro* and growth *in vivo*, as well as increased Wnt signaling. By contrast siRNA mediated knockdown of GATA6 led to corresponding decreases in these same parameters. The effects of GATA6 were found to be due to its ability to bind DNA, as forced overexpression of a DNA-binding mutant of GATA6 had no effects on cell growth *in vitro* or *in vivo,* nor did they affect Wnt signaling levels in these same cells. A microarray analysis revealed the Wnt antagonist Dickopf-1 (DKK1) as a dysregulated gene in association with GATA6 knockdown, and direct binding of GATA6 to the DKK1 promoter was confirmed by chromatin immunoprecipitation and electrophoretic mobility shift assays. Transient transfection of GATA6, but not mutant GATA6, into cancer cell lines led to decreased DKK1 mRNA expression and secretion of DKK1 protein into culture media. Forced overexpression of DKK1 antagonized the effects of GATA6 on Wnt signaling in pancreatic cancer cells. These findings illustrate that one mechanism by which *GATA6* promotes pancreatic carcinogenesis is by virtue of its activation of canonical Wnt signaling via regulation of DKK1.

## Introduction

GATA6 is a member of the GATA transcription factor family that plays critical regulatory roles in tissue development [1]. GATA proteins share a conserved zinc finger sequence that binds to the canonical DNA motif (G/A)GATA(A/T) [2] and are divided into two subgroups based on spatial and temporal expression patterns. GATA1/2/3 are expressed in hematopoietic cell lineages, and GATA4/5/6 in mesoderm and endoderm derived organs [1], [3]. GATA6 in particular is essential for the development of the heart, gastrointestinal tract, pancreas and other tissues [4], [5]. The importance of GATA6 is underscored by the observation that targeted inactivation of the *GATA6* gene in mice causes early embryonic lethality as a result of a lack of endoderm differentiation [5]-[7].

Recurrent copy number gain of *GATA6* has recently been identified in pancreatic duct adenocarcinoma (PDAC) cell lines and xenografts [8], [9]. While its role in PDAC carcinogenesis is unknown, mounting evidence indicates that GATA6 is associated with tumorigenesis in a variety of tissue types [10]-[14]. In ovarian tumors, ectopic GATA6 expression is correlated with cell dedifferentiation [15] whereas in colorectal cancer, GATA6 influences cell proliferation and apoptosis by affecting the expression of 15-Lipoxygenase-1 that plays a role in p53-dependent cell arrest [16]. Aberrant GATA6 expression of has also been implicated in human adrenal tumors as well as in an alpha/SV40 T-antigen transgenic mouse model that develops adrenocortical tumors in a gonadotropin-dependent fashion [17], [18]. By contrast, *GATA6* has been implicated as a tumor suppressor gene in astrocytomas [14].

This study sought to clarify the mechanisms by which GATA6 contributes to pancreatic carcinogenesis. We now show that *GATA6* amplification occurs during the late stages of pancreatic intraepithelial neoplasia, is significantly correlated with patient outcome, and promotes pancreatic carcinogenesis by activating the canonical Wnt signaling pathway due to its direct transcriptional repression of the secreted Wnt antagonist Dickkopf-1.

## Methods

### Ethics Statement

All human tissue samples were collected with approval of the Johns Hopkins Hospital Institutional Review Board (IRB protocols # NA_00036610 and NA_00001584) after informed and written consent. For animal experiments, studies were carried out in strict accordance with the recommendations in the Guide for the Care and Use of Laboratory Animals of the National Institutes of Health. The protocol was approved by the Committee on the Ethics of Animal Experiments of the University of Minnesota (ACUC protocol # MO09M84). All procedures were performed under sodium pentobarbital anesthesia, and all efforts were made to minimize suffering.

### Cell Lines and Tissues

The A6L, A13A, A10.7 and IMIM-PC2 cell lines were established in our own laboratories. PK8 and PK9 were from Dr. Akira Horii (Tohoko University, Sendai, Japan), and HCG-25 from Dr. Tony Hollingsworth (University of Nebraska Medical Center, Omaha NE). All remaining pancreatic cancer cell lines used were obtained from the ATCC (Manassas VA). The normal pancreatic duct epithelial cell lines HPNE and HPDE were prepared as previously described [19]. The colon cancer cell lines HCT116 (*CTNNB1* mutant), SW480 (*APC* mutant) and RKO (*APC* and *CTNNB1* wild type) were provided by Dr. James Eshleman (Johns Hopkins Medical Institutions, Baltimore MD USA). All cells were cultured in DMEM (Invitrogen, Carlsbad, CA, USA) supplemented with 10% fetal bovine serum (FBS), 100 units/ml penicillin, 100 mg/ml streptomycin and 2 mmol/L L-glutamine at 37°C and 5% CO_2_.

Human pancreatic tissue samples were obtained from the Surgical Pathology Department of Johns Hopkins and xenografts were generously provided by Dr. Scott Kern (Johns Hopkins Medical Institutions, Baltimore MD USA). All samples were collected with approval of the Johns Hopkins Hospital Institutional Review Board.

### Lentiviral Constructs

Recombinant lentiviruses expressing GFP downstream of a mock shRNA or an shRNA specific to GATA6 were created using a three-virus system as previously described in detail [20], [21]. Oligonucleotide sequences for GATA6 knockdown were sense, 5′-gatccgctgtcacaccacaactaccttcaagagaggtagttgtggtgtgacagctttttta-3′; and anti-sense, 5′-cgataaaaaagctgtcacaccacaactacctctcttgaaggtagttgtggtgtgacagcg-3′. Following infection, an aliquot of each cell line (1×10^5^) was analyzed by FACS in the Flow Cytometry Core Facility to confirm the efficiency of transduction (>95% in all cell lines tested) by monitoring GFP expression 60 h after transduction.

### Plasmid Constructs

The human GATA6 vector pcDNA3.1-GATA6 was a gift from Dr. Clement Ho (University of Pennsylvania, Philadelphia PA) and used to create pcDNA3.1-mGATA6 by site directed mutagenesis (Invitrogen) in which the eight most highly conserved bases of the zinc-finger motif were deleted [22], [23]. Human DKK1 expression vector pCMV-DKK1 and the DKK1 promoter luciferase reporter pGL3-DKK1 were both generously provided by Dr. Hirotaka Osada (Aichi Cancer Instititue, Nagoya, Japan). Stable cell lines were created following our methods previously reported in detail [8].

### In vitro Assays of Cell Growth

Cell proliferation assays were performed using Cell Counting Kit-8 (Dojindo Molecular Technologies) following the suggested protocol. Colony formation assays were performed as previously described [8]. All assays were performed in triplicate.

### Cell Cycle Analysis

Flow cytometry was performed on a Becton Dickenson LSR Benchtop Flow Cytometer (BD Biosciences, San Jose, CA). Percentages of cells in G0-G1, S, and G2 phase were determined using CellQuest (BD Biosciences).

### Enzyme Linked Immunosorbent Assay

ELISAs were performed using mouse mAb anti-human DKK-1 antibody (Clone 141119) following the manufacturers protocol (R & D Systems, Inc., Minneapolis, MN, USA). All assays were performed in triplicate.

### Western Blotting

Equal amounts of protein were separated on 15% SDS-polyacrylamide and transferred onto PVDF membranes (DuPont NEN, Boston, MA). Membranes were hybridized with a 1:100 dilution of primary antibody (ß-catenin mouse mAb clone E-5 or GATA6 rabbit pAb clone H-92, Santa Cruz Biotechnology, CA) followed by horseradish peroxidase (HRP)-linked goat anti-rabbit IgG and visualized by the enhanced chemiluminescence (ECL) system (Amersham). Expression of ß-actin was used as an internal control.

### Immunohistochemistry

Immunolabeling was performed following previously reported methods standard methods [8] using a 1∶100 dilution of each primary antibody for 2 hours at room temperature. The specificity of antibodies to GATA6 and DKK1 is presented by full-screen Western blotting in Figure S1.

### Gene Expression Microarrays

Total RNA was isolated with an RNeasy kit (Qiagen, Valencia, CA). Samples were hybridized to Agilent Human 4×44K arrays (Santa Clara, CA) and the raw data analyzed following standard protocols in the Microarray Core Facility at Johns Hopkins. All data is MIAME compliant and the raw data files have been deposited in the MIAME compliant GEO database (*accession number* GSE27173).

### Real Time Quantitative PCR for mRNA Expression

One microgram of RNA per sample was reverse transcribed into cDNA using SuperScriptTMIII Platinum® Two-Step qRT-PCR Kit (Invitrogen). RT-qPCR analysis was performed using a 7300 Real Time PCR System (Applied Biosystems, CA, USA) for monitoring of green dye fluorescence (SYBR®Green, Invitrogen Inc, CA, USA). Relative fold-changes of gene expression compared to the housekeeping gene b-actin were determined by calculation of the 2^ΔΔCt^. All assays were performed in triplicate. (Primer sequences are provided in File S1).

### GATA6 Copy Number Assays

Genomic DNA copy number of GATA6 was determined as described previously in detail [8]. Copy numbers of >2.3 were considered copy number gain to account for polysomy of chromosome 18q, and because up to 20-fold GATA6 overexpression may occur in PDACs with even low level copy number gain [8].

### Luciferase and TOPFLASH Assays

For TOPFLASH assays, pGL3-OT and pGL3-OF plasmids were used (kindly provided by Dr. Bert Vogelstein). Relative Wnt activity was determined by the ratio of luciferase expression from the pGL3-OT vector divided by that of the pGL3-OF vector as previously described. For all other luciferase assays, the vector pRL-TK (Promega) expressing sea pansy luciferase was used as a control. The pcDNA3.1-empty vector was used to adjust the total amount of transfected DNA. Luciferase assays were performed 40 hrs after transfection using the Dual-Luciferase-Reporter Assay System (Promega), and luciferase activity was determined with the 1420 multilabel counter (PerkinElmer life and analysis science, CT, USA). Firefly luciferase activities were normalized by sea pansy luciferase activities. All experiments were carried out in triplicate.

### Chromatin Immunoprecipitation Assay (CHIP assay)

ChIP assays were performed using reagents and protocols from Upstate Biotechnology (Lake Placid, NY) using previously described methods [24]. All primers used were designed to specifically target GATA binding motifs in the *DKK1* promoter. (Probe sequences are provided in File S1).

### Electrophoretic Mobility Shift Assay (EMSA)

EMSA assays were performed using previously described methods [24]. Protein Extracts were normalized for total protein, and 5–10 mg of protein were incubated with the ^32^P-labeled high-affinity GATA6 probes specific to each of the four putative GATA binding motifs within the *DKK1* promoter. Mutant probes in which the binding motif sequence was mutated were also used. A GATA6-P probe (5′-GCCAGCAGATAGCATGGAAAAG-3′) derived from *TFF2* promoter containing a GATA6 binding site [25] was used as a positive control. Protein-DNA complexes were resolved on 5% nondenaturating polyacrylamide gels and analyzed by autoradiography using Kodak film. (Probe sequences are provided in File S1).

### shRNA Mediated Knockdown

Cultured cells in log phase growth (50% confluent) were transfected with DKK1 shRNA (Dharmacon), CTNNB1 shRNA (CTNNB1-VHS50819, Invitrogen Inc, CA) or mock shRNA (#4611, Ambion) using Lipofectamine RNAiMAX (Invitrogen Inc, CA) following the recommended protocol. Twenty-four hours after transfection, cells were harvested and subjected to RT-qPCR analysis, cell proliferation and colony formation assays.

### Flourescent in situ hybridization (FISH)

FISH was performed as described previously [8] using bacterial artificial chromosome clones CTD-2376C8 containing the genomic sequences of the 18q11.2 amplicon at 0.11 Mb (Invitrogen, Carlsbad, CA).

### Methylation Specific PCR

Promoter methylation was assessed using primers and conditions previously reported by Suzuki et al [26].

### Statistical analysis

Statistical analyses were performed with an unpaired *t*-test for parametric distributions, or a Chi-squared test for comparing frequencies. P<0.05 was considered statistically significant.

## Results

### GATA6 Copy Number Gain Occurs during Pancreatic Intraepithelial Neoplasia

**Figure 1:**
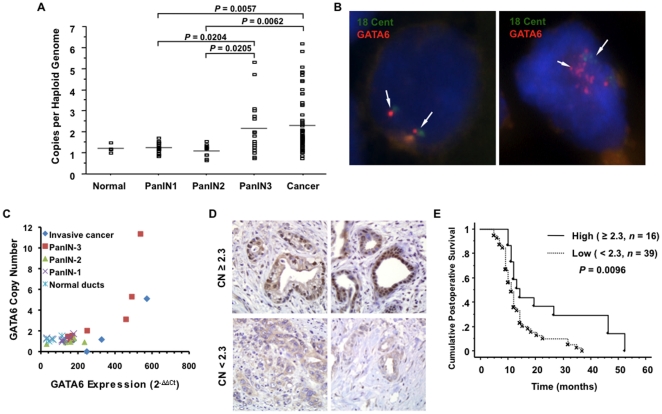
*GATA6* Copy Number Gain Correlates with Intraductal Progression of Pancreatic Cancer. (**A**) *GATA6* copy number (mean ± SE) in microdissected normal ducts (N = 4), PanIN-1 (N = 13), PanIN-2 (10), PanIN-3 (N = 17) lesions and pancreatic cancer (N = 55). (**B**) Representative FISH of the nucleus of a neoplastic cell within a PanIN3 lesion with >11-fold *GATA6* amplification (right) compared to the nucleus of a neoplastic cell from a different PanIN3 lesion without copy number gain of *GATA6* (left). GATA6 probe was labeled with red and chromosome 18 centromere probe (18 Cent) was labeled with green. The sections were counterstained with DAPI to highlight nuclei. (**C**) Correlation of GATA6 mRNA expression and copy number in microdissected samples of normal, PanIN and cancer tissue. (D) GATA6 immunolabeling of two pancreatic cancer tissues with GATA6 copy number gain compared to two cancers without copy number gain. Increased copy number is highly associated with nuclear labeling of GATA6 protein. (**E**) Kaplan Meier survival curve illustrating the relationship of *GATA6* copy number gain (≥2.3 copies per haploid genome) to overall survival in patients with surgically resected pancreatic cancer.

Normal pancreatic ductal epithelium is believed to progress to infiltrating cancer through a series of morphologically defined precursors called pancreatic intraepithelial neoplasia (PanIN-1, 2, 3) [27]. To understand the correlation between genetic gain of *GATA6* and PDAC development, we assessed *GATA6* copy number in microdissected samples of normal duct epithelium, PanIN, and human PDAC by quantitative PCR. Relative to the haploid genome, there was no gain of *GATA6* in normal duct epithelium (0 of 4), PanIN-1 (0 of 13) or PanIN-2 (0 of 10) lesions. By contrast, increased *GATA6* copy number (≥2.3 copies) was identified in 6/17 samples (35%) of PanIN-3 and in 18/55 samples (33%) of PDAC (Figure 1A). *GATA6* copy number gain was further confirmed by fluorescent *in situ* hybridization (FISH) in paraffin-embedded sections of one PanIN-3 and 10 PDAC samples (Figure 1B).

For six patients the matched PanIN-3 and PDAC were microdissected from the same tissue section and analyzed for *GATA6* copy number, *KRAS* and *TP53* gene status (Table 1). In two patients (patients 7 and 53) *GATA6* copy number gain was found in both the PanIN-3 and PDAC samples indicating it arose prior to the development of infiltrating carcinoma, whereas in a third patient (patient 53) *GATA6* copy number gain was only present within the PDAC sample suggesting it arose during temporal progression to PDAC. However, as a single PanIN-3 was analyzed in this patient, we cannot rule that additional and untested PanIN-3 lesions in this patient's pancreas also contained copy number gain. To confirm that increases in *GATA6* copy number result in increased gene expression, we quantified GATA6 mRNA levels in these same microdissected samples (Figure 1C), indicating that relative levels of GATA6 mRNA were significantly greater in samples with *GATA6* copy numbers ≥2.3 compared to those with copy numbers <2.3 (461.9±126.2 and 194.1±69.7, p<0.0004). Similarly, immunolabeling for GATA6 in five pancreatic cancers with copy number gain showed strong positive nuclear labeling whereas no labeling was seen in five pancreatic cancers with copy numbers <2.3 (Figure 1D).

**Table 1:** Genetic Features of Matched Pancreatic Intraepithelial Neoplasia and Cancer Samples.

Patient	Histology	GATA6 Copy Number	KRAS	TP53
7	PanIN-3	5.3	G12R	R248W
	PDAC	5.1	G12R	R248W
10	PanIN-3	3.11	G12V	13 bp del 288
	PanIN-3	2.75	G12V	13 bp del 288
	PDAC	0.88	G12V	13 bp del 288
53	PanIN-3	4.72	nd^a^	G245S
	PDAC	3.82	nd^a^	nd^a^
58	PanIN-3	0.81	G12D	1 bp insert 255
	PDAC	2.38	G12D	1 bp insert 255
64	PanIN-3	1.36	G12D	WT
	PDAC	1.04	G12D	WT
68	PanIN-3	1.14	G12V	WT
	PDAC	1.16	G12V	WT

a Not determined.

Pancreatic carcinogenesis is accompanied by the accumulation of genetic alterations in the *KRAS, CDKN2A, TP53* and *SMAD4* genes [27]. We therefore determined the relationship of *GATA6* copy number gain to the genetic status of these four genes in 56 xenograft enriched PDACs. Seventeen xenografts (30%) had a *GATA6* copy number ≥2.3 relative to the haploid genome, of which six (11%) had a copy number >5.0. However, there was no correlation of *KRAS, CDKN2A, TP53* or *SMAD4* status with *GATA6* copy number. Because *GATA6* is also located on the same chromosome arm as *SMAD4* that is frequently targeted by homozygous deletion [28], we next wondered if *GATA6* copy number gain is specifically related to genomic rearrangement events that may lead to homozygous deletion of *SMAD4* in the same xenograft DNA. However, this again did not reveal an association, with six of eight *SMAD4* mutants occurring due to homozygous deletion in xenografts with increased *GATA6* copy number versus 13 of 21 with a homozygous deletion in the xenografts without *GATA6* copy number gain (p = 0.2844). Taken together, we conclude that *GATA6* copy numbe≥r gain occurs during late stages of pancreatic intraepithelial neoplasia but is not specifically enriched for within carcinomas with alterations of these four genes.

We next determined the extent to which *GATA6* copy number gain is associated with clinicopathologic features of resected PDAC. No relationships were found for *GATA6* and age, gender, tumor size, tumor differentiation, tumor location or lymph node status. However, patients with copy number ≥2.3 had a longer overall survival than patients without copy number gain by Kaplan Meier survival estimate (p = 0.0096, Figure 1E).

### GATA6 Promotes Cell Growth *In Vitro* and *In Vivo*

**Figure 2:**
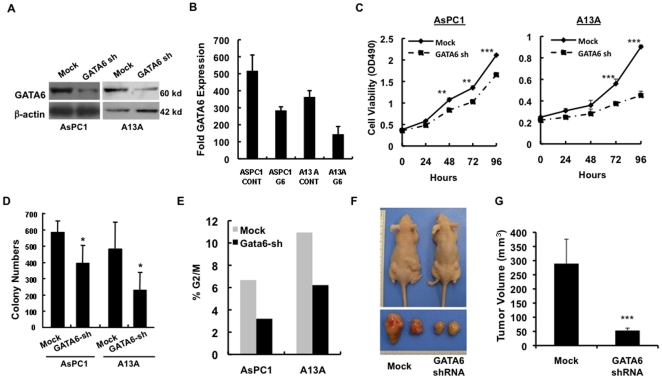
Effects of GATA6 Knockdown on Cell Growth *in vitro* and *in vivo*. (**A**) Total protein was extracted from AsPC1-GATA6sh and A13A-GATA6sh cells and mock shRNA controls and analyzed by Western blot for relative levels of GATA6 protein relative to actin. (**B**) Real-time PCR for GATA6 expression in AsPC1-GATA6sh and A13A-GATA6sh cells. (**C**) These cells were also analyzed for cell proliferation at different time points, (**D**) cultured in soft agar and the number of colonies at 2 weeks counted, and (**E**) analyzed by flow cytometry to determine the percent of cells in G2/M phase. (**F**) Representative xenograft formation *in vivo* (above) and after explantation (lower) of AsPC1 control and GATA6sh cells at 8 weeks postinjection. (**G**) Average tumor volume (mean ± SE) of these same xenografts at 8 weeks postinjection. Similar results were noted for A13A-GATA6sh cells (data not shown). With exception of flow cytometry that was performed in duplicate, all experimental data shown represents the summary three independent experiments. *, p<0.05; **, p<0.01; ***, p<0.001.

**Figure 3:**
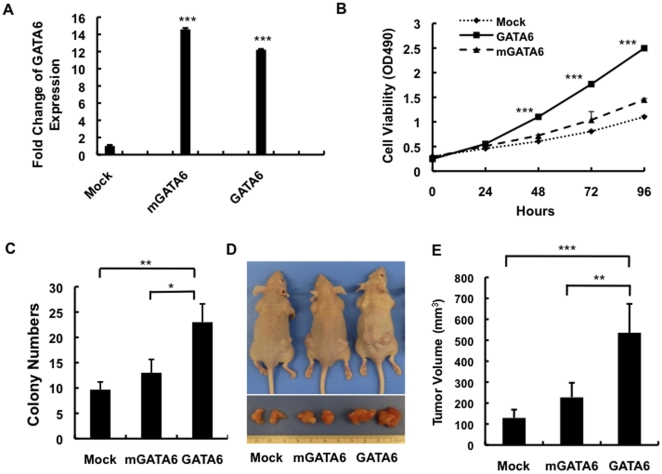
Effects of GATA6 Overexpression on Cell Growth *in vitro* and *in vivo*. (**A**) Real-time PCR for GATA6 expression in Panc1-mock, Panc1-GATA6 and Panc1-mGATA6 cells. (**B**) Panc1-mock, Panc1-GATA6 and Panc1-mGATA6 cells were either analyzed for cell proliferation or (**C**) cultured in soft agar and the number of colonies at 2 weeks counted. (**D**) Representative xenograft formation *in vivo* (above) and after explantation (lower) of Panc1-mock, Panc1-GATA6 and Panc1-mGATA6 cells at 8 weeks postinjection. (**E**) Average tumor volume (mean ± SE) of these same xenografts at 8 weeks postinjection. All experimental data shown represents the summary three independent experiments. *, p<0.05; **, p<0.01; ***, p<0.001.

Amplification and overexpression of *GATA6* in PanIN and PDAC suggests it contributes to PDAC biology [8], [9]. We therefore constructed lentiviral vectors that express either a mock or GATA6 specific shRNA and used them to stably infect the PDAC cell lines AsPC1 and A13A with copy numbers of 2.3 and 9.0 relative to the haploid genome, respectively [8], [9]. In both cell lines, GATA6 is also overexpressed at least 10-fold relative to normal duct cells [8], [9] (Figure S2). Knockdown of GATA6 in both cell lines (Figure 2A, 2B) led to significant decreases in cell proliferation and colony formation (Figure 2C and D), a reduction in cells within G2/M phase (Figure 2E) and decreased growth in vivo (Figure 2F and 2G). Conversely, forced overexpression of GATA6 in the PDAC cell line Panc1 with low levels of endogenous GATA6 expression [8] (Figure 3A and Figure S2) led to increased cell proliferation and colony formation (Figure 3B and 3C) similar to that also previously shown for the cell line MiaPaca2 that does not have endogenous expression of GATA6 [8].

GATA6 regulates DNA transcription by binding to canonical GATA motifs, or as a transcriptional cofactor [2], [29]. To determine if the effects in Panc1 cells were due to GATA6 DNA binding activity, we used site-directed mutagenesis to create a mutant cDNA in which the GATA6 Zn finger domain was disrupted (called mGATA6), and again stably transfected Panc1 cells (Figure 3A). There was no difference in cell growth or colony formation among Panc1-mGATA6 cells and Panc1-control transfected cells (Figure 3B and 3C) suggesting that the growth promoting effects of GATA6 observed *in vitro* are due to its function as a transcription factor. To further clarify the growth-promoting effect of GATA6, nude mice were inoculated subcutaneously with Panc1-GATA6 or Panc1-mGATA6 cells. After 8 weeks, the mean tumor volume was significantly larger in mice injected with Panc1-GATA6 cells than with Panc1-mGATA6 cells (Figure 3D and 3E), leading us to conclude that GATA6 promotes carcinogenesis via its ability to bind DNA.

### The Wnt Antagonist Dickkopf1 (DKK1) is a GATA6 Target Gene

**Figure 4:**
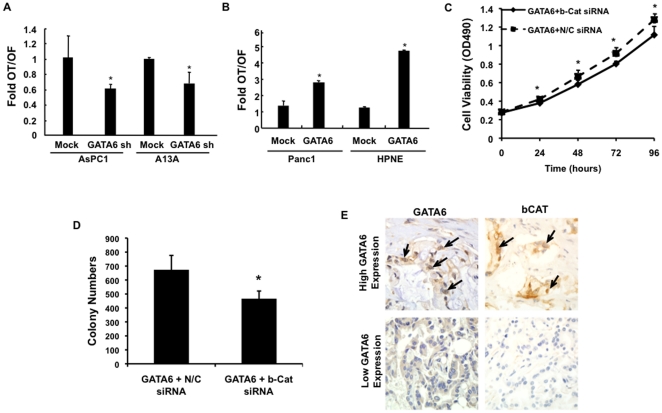
GATA6 Overexpression Correlates with Canonical Wnt Signaling. (**A**) Wnt signaling activity in AsPC1-GATA6sh and A13A-GATA6sh cells based on TOPFLASH assay. Luciferase activity is represented as the ratio of OT to OF levels in cells with GATA6 knockdown relative to that of mock-transfected cells. (**B**) Wnt signaling activity in Panc1-GATA6 and HPNE-GATA6 cells determined by TOPFLASH assay. Luciferase activity is represented as the ratio of OT to OF levels in GATA6 transfected cells relative to that of mock-transfected cells. (**C and D**) Panc1-GATA6 cells were transiently transfected with ß-catenin or mock shRNA and (**C**) cell proliferation or (**D**) colony formation determined. All experimental data shown represents the summary three independent experiments. *, p<0.05; **, p<0.01. (E) Immunolabeling patterns of GATA6 and ß-catenin protein in two representative PDAC tissues. Arrows indicate nuclear labeling of both GATA6 and ß-catenin in serial sections of the same cancer tissue. By contrast, the PDAC sample with low GATA6 expression also shows no expression of ß-catenin.

GATA proteins are linked to Wnt signaling in embryogenesis of the heart and lungs [4], [30], [31]. We therefore hypothesized that GATA6 contributes to pancreatic carcinogenesis in part through its effects on Wnt signaling, a putative relationship that has not been explored in any detail for this tumor type. Compared to mock shRNA lentiviral-infected cells, both AsPC1-GATA6sh and A13A-GATA6sh cells showed a significant decrease in functional Wnt signaling activity by TOPFLASH assay (Figure 4A) whereas overexpression of GATA6 in Panc1 and HPNE cells promoted Wnt signaling activity (Figure 4B). To determine if ß-catenin expression is required for these effects we silenced ß-catenin expression using an shRNA strategy in Panc1-GATA6 cells, leading to a significant inhibition of cell proliferation and colony formation (Figure 4C and 4D). In pancreatic cancer tissues, GATA6 overexpression was significantly correlated with nuclear accumulation of ß-catenin protein (8/12 PDACs with GATA6 overexpression showing ß-catenin nuclear accumulation versus 3/20 without GATA6 overexpression, p = 0.004) (Figure 4E). Thus, GATA6 overexpression in PDAC contributes to cell proliferation and colony formation by enhancing canonical Wnt signaling.

**Figure 5:**
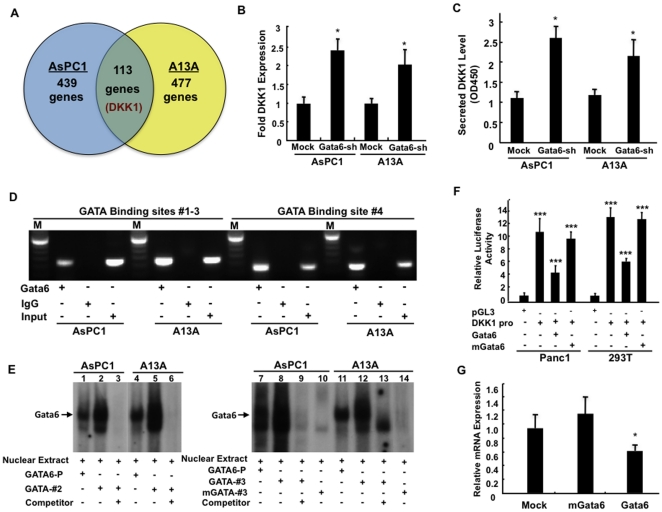
*DKK1* is a GATA6 target gene. (**A**) Venn diagram indicating the number of dysregulated genes identified by microarray analysis of AsPC1-GATA6sh (left circle, blue) and A13A-GATA6sh cells (right circle, yellow). The green cross-area indicates commonly dysregulated genes and includes DKK1. (**B**) Real-time PCR confirming DKK1 overexpression in AsPC1-GATA6sh and A13A-GATA6sh cells. (**C**) Detection of secreted DKK1 protein in conditioned media in AsPC1-GATA6sh and A13A-GATA6sh cells. Secreted DKK1 protein levels are indicated by using absorbance OD450. (**D**) Chromatin immunoprecipitation assay confirming binding of GATA6 to the *DKK1* promoter. Non-immune IgG and whole genome derived gDNA are used as negative and positive controls, respectively. (**E**) EMSA assay confirming binding of GATA6 to putative GATA binding sites #2 and #3. The mutant sequence mGATA-#3 did not generate any detectable binding. Nuclear Extr, nuclear extract; GATA6-P, a positive control probe derived from the *TFF2* promoter containing a GATA6 binding site; GATA-#2, probe containing putative GATA binding site No. 2; GATA-#3, probe containing putative GATA binding site No. 3; mGATA-#3, probe containing a mutated putative GATA binding site No. 3; refer to methods for additional details (**F**) Effect of GATA6 expression on activity of the *DKK1* promoter. Data is presented as the ratio of firefly luciferase activity to sea pansy luciferase activity. pGL3 was used as a negative control for background. (**G**) Real-time PCR for DKK1 mRNA expression in Panc1 cells. When appropriate, all experimental data shown represents the summary three independent experiments. *, p<0.05; ***, p<0.001.

GATA6 regulates its target genes through binding to the GATA-binding motif [2]. To identify GATA6 target genes that may influence Wnt signaling, we performed gene expression profiling using AsPC1-GATA6sh and A13A-GATA6sh and their mock controls and identified 113 commonly dysregulated genes (Figure 5A and Table S1) one of which was Dickkopf-1 (*DKK1*), an antagonist of canonical Wnt signaling [32]. Wnt11, a known GATA6 target gene [30], was also identified suggesting that GATA6 contributes to Wnt pathway regulation in part through regulation of these genes. Because DKK1 has not previously been recognized as a *GATA6* target gene we specifically focused on the relationship of GATA6 to DKK1.

Real-time PCR confirmed DKK1 mRNA upregulation and increased secretion of DKK1 protein into cell media in both cell lines in the presence of GATA6 knockdown (Figures 5B and 5C). To determine if *DKK1* is a direct target of GATA6, we searched the *DKK1* promoter for GATA6 consensus binding sequences. Four independent GATA-binding motifs were identified (Figure S3), and by chromatin immunoprecipitation assay direct binding of GATA6 to these motifs within the *DKK1* promoter was demonstrated (Figure 5D). Binding by GATA6 was also confirmed by electrophoretic mobility shift assay (Figure 5E and data not shown). We next used a luciferase reporter under control of the *DKK1* promoter to determine if GATA6 binding to *DKK1* impacts upon transcriptional activity from the gene. This reporter was activated in both 293T and Panc1 cells reflecting endogenous activation of DKK1 expression. However, upon forced GATA6 expression (Fig. 5F) luciferase activity was significantly decreased, whereas no effect on *DKK1* promoter activity was seen in the presence of the GATA6 binding motif mutant (mGATA6), indicating that the repressive effects of GATA6 on *DKK1* requires direct binding of GATA6 to the *DKK1* promoter. Forced expression of wild-type but not mGATA6 protein in Panc1 also led to significant decreases in DKK1 mRNA levels (Figure 5G). Taken together, these data indicate that GATA6 negatively regulates DKK1 transcription through direct binding to the GATA motif in the *DKK1* promoter region.

### DKK1 Expression in PDAC Correlates with Wnt Activation

**Figure 6:**
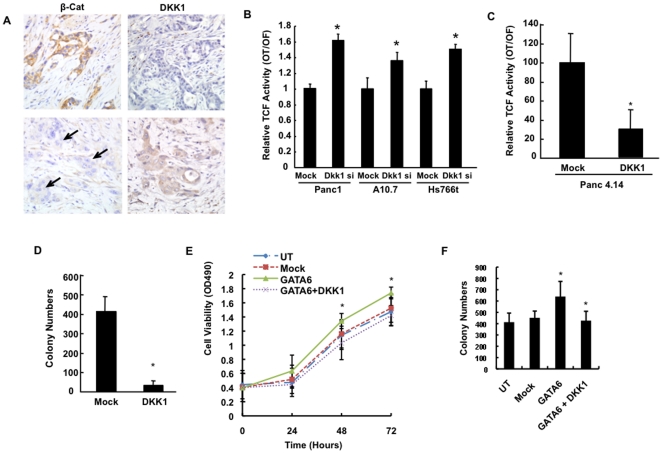
DKK1 Mediates GATA6 Effects on Wnt Signaling. (**A**) Representative DKK1 and ß-catenin immunolabeling in two human pancreatic cancer tissues. Arrows in the bottom left panel indicate location of cancer cells with negative labeling for ß-catenin (all images x400) (**B**) Panc1, A10.7 and Hs766t cells were transfected with a shRNA against DKK1 and subjected to TOPFLASH assay. Wnt activities are presented as a ratio of OT activity to OF activity. (**C and D**) Panc 4.14 cells were transfected with a DKK1-expressing vector and subjected to (**C**) TOPFLASH assay or (**D**) cultured in soft agar and the number of colonies at 2 weeks counted. Panc1 cells were transfected with mock, GATA6, and/or DKK1 expressing vectors, and cells were analyzed for (**E**) cell proliferation, or (**F**) cultured in soft agar and number of colonies at two weeks counted. When appropriate, all experimental data shown represents the summary three independent experiments. *, p<0.05.

Members of the DKK family (DKK1, DKK2, DKK3 and DKK4) are secreted proteins that inhibit canonical Wnt signaling by binding to a subunit of the Wnt receptor complex LRP5/6 [32]. Real-time PCR indicated that DKK1 was the only member of the DKK family that was expressed in both normal duct cell lines and in the majority of PDAC cell lines analyzed (Figure S4A). Immunohistochemical labeling for DKK1 showed at least focal labeling in 27/32 (84%) PDAC tissues (Figure 6A), although in only 3 cases was strong intensity labeling seen. DKK1 showed low levels of expression in the 12 PDAC tissues with GATA6 overexpression consistent with GATA6 repression. However, DKK1 was only overexpressed in 3/20 PDAC tissues without GATA6 expression, suggesting cellular mechanisms in addition to GATA6 may repress DKK1 expression in PDAC. Therefore, to determine if epigenetic silencing plays a role in DKK1 expression we performed methylation specific PCR of the *DKK1* promoter region (Figure S4B). Five of seven PDAC cell lines with low levels of DKK1 mRNA expression had promoter methylation of *DKK1*, and four of these five also expressed low levels of GATA6, confirming the possibility that DKK1 epigenetic silencing occurs in a subset of PDACs that do not overexpress GATA6 as described for other tumor types [26], [33].

To determine the extent to which DKK1 expression affects canonical Wnt signaling in pancreatic cancer, we silenced DKK1 expression in Panc1, Hs766t and A10.7 cells with high endogenous levels of DKK1 expression (Figures S4A and S5A). DKK1 knockdown significantly enhanced the activity of a TOPFLASH reporter (Figure 6B) and promoted cell proliferation (Figure S5B). By contrast, forced overexpression of DKK1 in Panc 4.14 cells displaying high OT/OF activity and lacking endogenous DKK1 expression (Figures S4A and S5C) led to significantly decreased TOPFLASH reporter activity (Figure 6C), and significant reductions in colony formation (Figure 6D) suggesting that loss of DKK1 expression promotes human PDAC carcinogenesis through activation of canonical Wnt signaling. Because GATA6 is a direct transcriptional repressor of *DKK1* (Figure 5), we determined if Wnt signaling activation by GATA6 is specifically mediated by DKK1. Accordingly, forced co-expression of DKK1 in Panc1-GATA6 cells blocked the effects of GATA6 compared to Panc1-GATA6 cells challenged with a control vector (Figure 6E and 6F).

## Discussion

Pancreatic ductal adenocarcinoma is a genetically complex disease characterized by the accumulation of genetic alterations and by extensive genomic and transcriptomic alterations leading to cell cycle deregulation, cell survival, invasion and metastasis [34]. We now provide compelling evidence that *GATA6* copy number gain is an additional and recurrent genetic alteration to be considered for this tumor type, and contributes oncogenic signals by virtue of its enhancement of Wnt signaling.

Although Wnt signaling is aberrantly activated in PDAC [35], [36], mutations of *CTNNB1*, *APC* or other pathway components are rare in this tumor type suggesting alternative mechanisms for Wnt activation. At least two additional pathways known to be active in PDAC have also been reported as converging on the Wnt pathway [37], [38]. For example, a recent study has reported that ataxia-telangiectasia group D complementing gene (ATDC) has oncogenic potential through stabilizing ß-catenin and activating the Wnt pathway in PDAC [38] whereas in a mouse model of pancreatic carcinoma activation of Hedgehog signaling led to a corresponding activation of Wnt signaling in part due to upregulation of TCF4 expression [37]. This is consistent with our data as well as we demonstrate Wnt pathway activation due to transcriptional repression by GATA6 of the secreted Wnt antagonist DKK1. At the very least, the upregulation of Wnt activity mediated by GATA6 dependent repression of DKK1 further supports the view that GATA6 is an oncogene in pancreatic ductal adenocarcinoma.

Although *GATA6* amplification has been reported in pancreatic cancer cell lines and xenografts [8], [9], when *GATA6* amplification occurs during the step-wise progression of pancreatic intraepithelial neoplasia has not. We now show that GATA6 amplification occurs during the late stages of pancreatic intraepithelial neoplasia, specifically PanIN-3. Amplification lead to increased expression in these same lesions, and parallels prior observations GATA6 protein overexpression in pancreatic intraepithelial neoplasia and cancer [8], [9]. Perhaps the most important implication of this finding is that detectable *GATA6* copy number gain may have value as a diagnostic marker of PDAC while still in the curative stage, as this remains a critical hurdle to improving survival of this disease [39]. However, as genetic gain of *GATA6* was only identified in a third of samples, a more universal and sensitive marker of canonical Wnt signaling dysregulation may be of value for this purpose.

We also noted a significant relationship among *GATA6* copy number and overall survival, in that patients whose resected cancers had a copy number ≥2.3, or nuclear overexpression, had a longer overall survival than those patients without copy number gain. Although the biologic significance of this finding remains to be discerned, it is conceivable that PDACs with high *GATA6* copy number, and/or simply with active canonical Wnt signaling for which *GATA6* copy number is a marker, cosegregate with those PDACs with less aggressive features as we have recently shown for E-cadherin [40]. It is also important to note that proliferative rates alone do not fully represent aggressive biology (metastasis), an interpretation that is in keeping with our published data that pancreatic cancers with low metastatic ability (<10 metastases at autopsy) are actually larger at diagnosis, are more invasive into surrounding tissues than those with highly metastatic ability, yet are often associated with a longer overall survival [41]. In addition to its role in Wnt signaling, ß-catenin is also a critical component of the adherens junction complex that includes E-cadherin, p120, plakoglobin and γ-catenin. Binding of ß-catenin to γ-catenin links the adherens junction complex to the actin cytoskeleton, thereby providing mechanical stability [42], whereas disruption of this complex is associated with epithelial-mesenchymal transition and aggressive features in a variety of tumor types, including PDAC [43]. One interpretation is that the presence of canonical Wnt pathway activity together with an intact zona-adherens in PDAC, both that are dependent on ß-catenin expression, may be linked to less aggressive features than when ß-catenin expression is lost [40].

DKK1 belongs to the Dickkopf family comprised of four members that include DKK2, DKK3 and DKK4. With exception of DKK3, all members have the ability to modulate Wnt signaling through inhibiting the Wnt co-receptor LRP5/6 at the cell surface leading to destabilization of ß-catenin and its subsequent degradation [44]. Quantitative analysis indicated that DKK1 was the predominant member of this gene family expressed in PDAC cell lines, consistent with Takahashi et al [45]. Although that study did suggest DKK1 upregulation functions as an oncogene in PDAC cells, it is important to note that only Suit-2 and a related cell line were used in that study that was not used in the current work, and the levels of GATA6 in Suit-2 are unknown. By contrast, our data has relied on multiple cell lines selected based on GATA6 and DKK1 expression levels and Wnt signaling levels. Therefore it is conceivable that the conclusions drawn based use of the Suit-2 cell line, while valid, are not fully representative of DKK1 regulation in PDAC.

In summary, *GATA6* contributes to PDAC through activation of the canonical Wnt signaling pathway. This finding expands upon recent observations of *GATA6* amplification, and provides fertile ground for additional studies of the role of GATA transcription factors and Wnt signaling in this pathogenesis and aggressiveness of this tumor type.

## Supporting Information

Figure S1 **Western Blotting for GATA6 and DKK1 in Pancreatic Cancer Cell Lines.** The specificity of antibodies against GATA6 and DKK1 is shown by full-screen Western blotting. Exposures of both 2 and 5 minutes are shown.
(TIF)

Figure S2 **RT-PCR for GATA6 expression in human normal and pancreatic cancer cell lines.** β-actin is used as a loading control for each sample.
(TIF)

Figure S3 **Structure of the human** ***DKK1*** **promoter region.** The human *DKK1* promoter has a TATA box near the transcription site (TSS) and four GATA binding motif within 1 kb upstream from TSS. *, putative GATA binding site No. 1 (reverse); #, putative GATA binding site No. 2 (forward); ▴, putative GATA binding site No. 3 (forward); ¢, putative GATA binding site No. 4 (reverse). TATA box is enclosed by box. Sequences for the primer sets that be used in CHIP assay were indicated in bold type. TSS, transcription start site; Primer F1 (forward) and R1 (reverse) for amplifying the region containing three GATA binding motifs No. 1, 2 and 3. Primer F2 (forward) and R2 (reverse) for amplifying the region containing one GATA binding motif No. 4. The same region also contains four TCF-binding sites: TBE1, TBE2, TBE3 and TBE4.
(TIF)

Figure S4 **Expression and Methylation of Dickkopf-1 in Pancreatic Cancer Cell Lines.** (a) Quantitative RT-PCR of DKK1-4 in immortalized normal and pancreatic cancer cell lines. All values are normalized to levels in HPNE. (b) Promoter methylation of DKK1 in immortalized normal and pancreatic cancer cell lines.Methylation is detected in cell lines MiaPaca2, Hs766t, Panc 4.14, PK8 and PK9. All primer sequences are provided in Supplemental Information.
(TIF)

Figure S5 **Effect of Dickkopf-1 on cell proliferation in Pancreatic Cancer Cell Lines.** Panc1, A10.7 and Hs766t cell lines that have relatively high DKK1 expression levels were transfected with a mock siRNA or DKK1 siRNA and then subjected to (**A**) real-time PCR for DKK1 expression in DKK1-knock down cells or (B) cell proliferation assays. (C) Real-time PCR for DKK1 expression in Panc 4.14 cells after transfection with a mock or DKK1 expression vector. When appropriate, all experimental data shown represents the summary three independent experiments. **, p < 0.01; ***, p < 0.001.
(TIF)

Table S1 **Genes commonly dysregulated in both A13A-GATA6sh and AsPC1-GATA6sh cells.** A13A and AsPC1 cells were infected with a lentivirus expressing a mock shRNA or shRNA against GATA6 and subjected to cDNA microarray assay. Microarray data was analyzed using GeneChip analysis software. All mRNA transcripts was calculated for the GATA6-knock down samples and compared with mock control. Genes that were commonly desregulated in both A13A and AsPC1 cells are shown. Data deposited in the GEO database (number GSE27173).
(DOC)

File S1 **Oligonucleotide Sequences Used in Current Study.**
(DOC)
