# Supplementary figures and images for: GATA6 Activates Wnt Signaling in Pancreatic Cancer by Negatively Regulating the Wnt Antagonist Dickkopf-1
Source: PLoS One. 2011 Jul 19;6(7):e22129. doi: 10.1371/journal.pone.0022129 (PMC3139620; doi:10.1371/journal.pone.0022129)

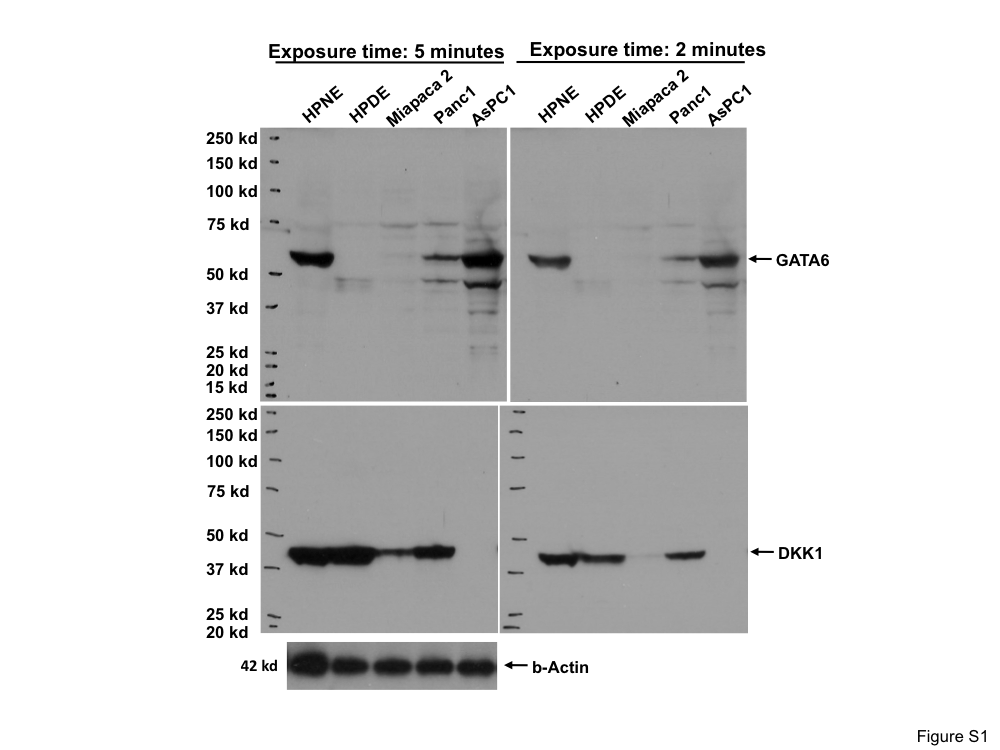

Supplement: Figure S1 — Western Blotting for GATA6 and DKK1 in Pancreatic Cancer Cell Lines. The specificity of antibodies against GATA6 and DKK1 is shown by full-screen Western blotting. Exposures of both 2 and 5 minutes are shown. (TIF) [file pone.0022129.s001.tif]

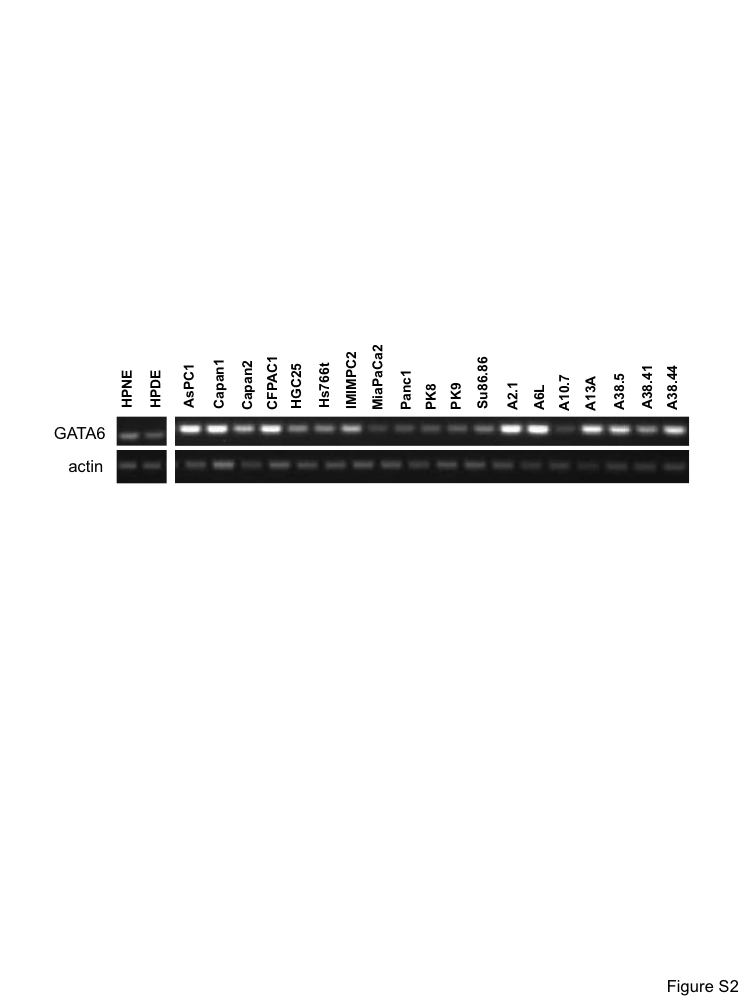

Supplement: Figure S2 — RT-PCR for GATA6 expression in human normal and pancreatic cancer cell lines. β-actin is used as a loading control for each sample. (TIF) [file pone.0022129.s002.tif]

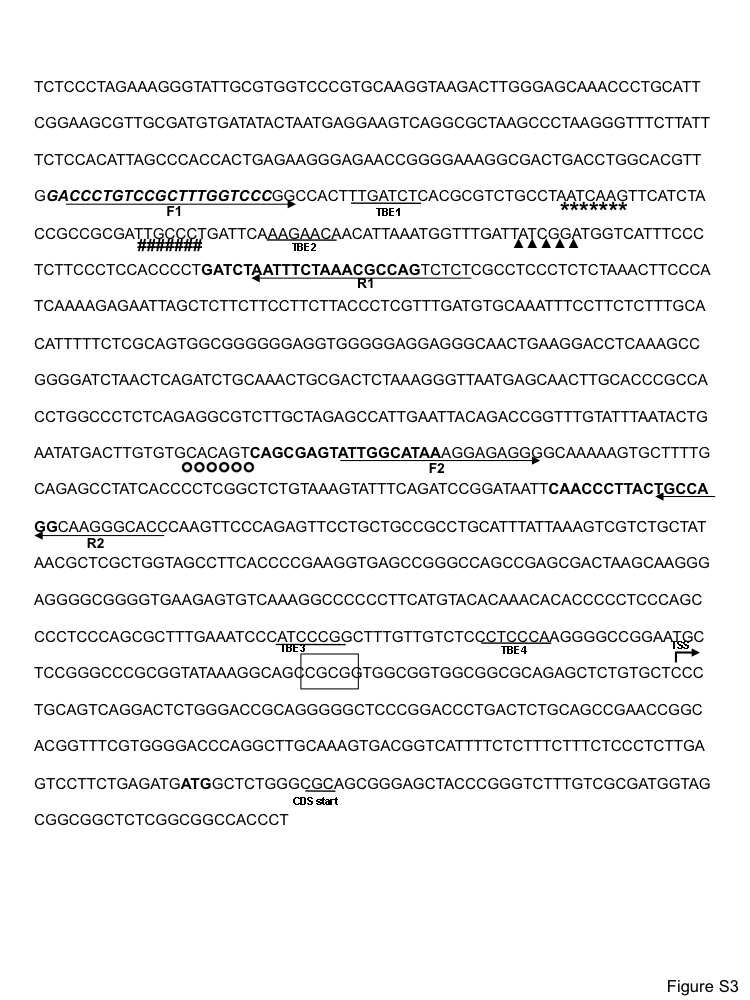

Supplement: Figure S3 — Structure of the human DKK1 promoter region. The human DKK1 promoter has a TATA box near the transcription site (TSS) and four GATA binding motif within 1 kb upstream from TSS. *, putative GATA binding site No. 1 (reverse); #, putative GATA binding site No. 2 (forward); ▴, putative GATA binding site No. 3 (forward); ¢, putative GATA binding site No. 4 (reverse). TATA box is enclosed by box. Sequences for the primer sets that be used in CHIP assay were indicated in bold type. TSS, transcription start site; Primer F1 (forward) and R1 (reverse) for amplifying the region containing three GATA binding motifs No. 1, 2 and 3. Primer F2 (forward) and R2 (reverse) for amplifying the region containing one GATA binding motif No. 4. The same region also contains four TCF-binding sites: TBE1, TBE2, TBE3 and TBE4. (TIF) [file pone.0022129.s003.tif]

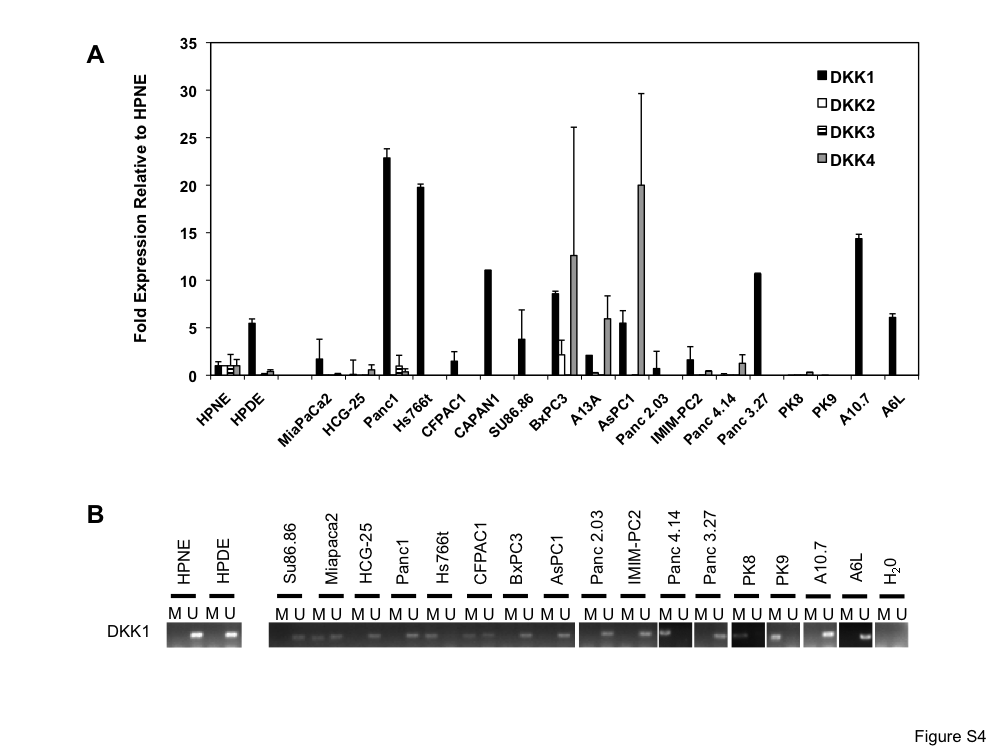

Supplement: Figure S4 — Expression and Methylation of Dickkopf-1 in Pancreatic Cancer Cell Lines. (a) Quantitative RT-PCR of DKK1-4 in immortalized normal and pancreatic cancer cell lines. All values are normalized to levels in HPNE. (b) Promoter methylation of DKK1 in immortalized normal and pancreatic cancer cell lines.Methylation is detected in cell lines MiaPaca2, Hs766t, Panc 4.14, PK8 and PK9. All primer sequences are provided in Supplemental Information. (TIF) [file pone.0022129.s004.tif]

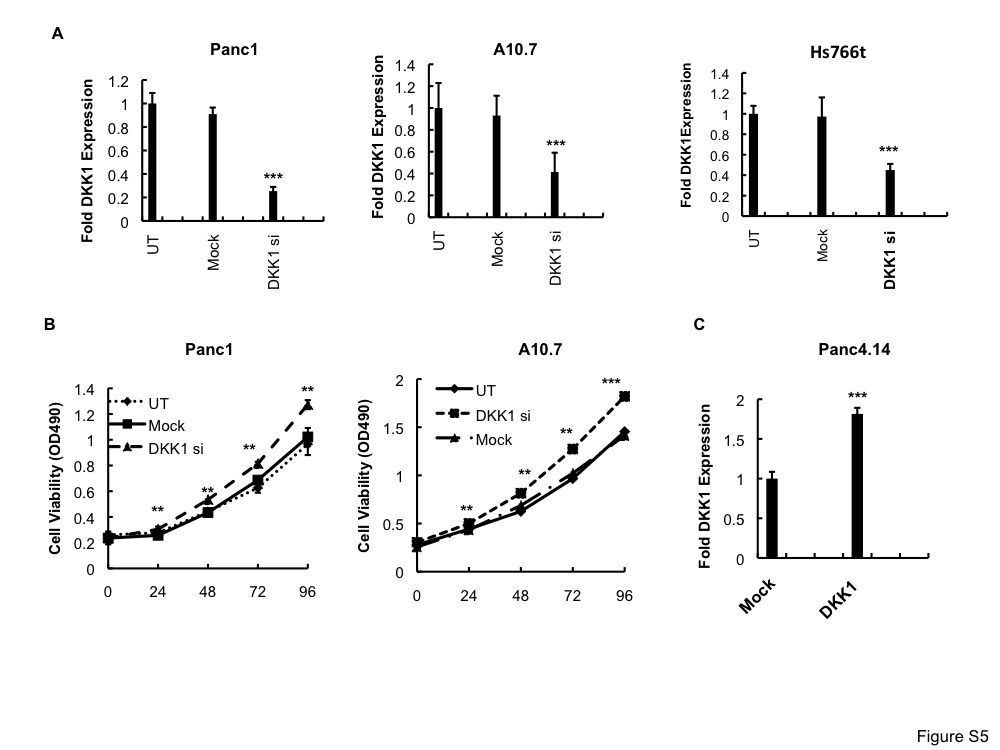

Supplement: Figure S5 — Effect of Dickkopf-1 on cell proliferation in Pancreatic Cancer Cell Lines. Panc1, A10.7 and Hs766t cell lines that have relatively high DKK1 expression levels were transfected with a mock siRNA or DKK1 siRNA and then subjected to (A) real-time PCR for DKK1 expression in DKK1-knock down cells or (B) cell proliferation assays. (C) Real-time PCR for DKK1 expression in Panc 4.14 cells after transfection with a mock or DKK1 expression vector. When appropriate, all experimental data shown represents the summary three independent experiments. **, p < 0.01; ***, p < 0.001. (TIF) [file pone.0022129.s005.tif]
