# Supplementary material for: GATA6 Activates Wnt Signaling in Pancreatic Cancer by Negatively Regulating the Wnt Antagonist Dickkopf-1
Source: PLoS One. 2011 Jul 19;6(7):e22129. doi: 10.1371/journal.pone.0022129 (PMC3139620; doi:10.1371/journal.pone.0022129)
Supplement: Table S1 — Genes commonly dysregulated in both A13A-GATA6sh and AsPC1-GATA6sh cells. A13A and AsPC1 cells were infected with a lentivirus expressing a mock shRNA or shRNA against GATA6 and subjected to cDNA microarray assay. Microarray data was analyzed using GeneChip analysis software. All mRNA transcripts was calculated for the GATA6-knock down samples and compared with mock control. Genes that were commonly desregulated in both A13A and AsPC1 cells are shown. Data deposited in the GEO database (number GSE27173). (DOC) [file pone.0022129.s006.doc]

| **Gene Symbol** | **Accession** | **Regulation** | **Fold change** | **Gene Symbol** | **Accession** | **Regulation** | **Fold change** |
| --- | --- | --- | --- | --- | --- | --- | --- |
| ALPPL2 | NM_031313 | down | 2.3151863 | ABCB9 | NM_019625 | up | 2.1367238 |
| APOL3 | NM_145641 | down | 2.301152 | AK021985 | AK021985 | up | 2.1019623 |
| ATAD4 | NM_024320 | down | 2.034353 | AK123649 | AK123649 | up | 2.0062964 |
| ATP11A | NM_032189 | down | 2.0006406 | AL567265 | AL567265 | up | 3.161332 |
| ATP2A3 | NM_174953 | down | 2.6663797 | ALDH5A1 | NM_170740 | up | 2.0312858 |
| BATF | NM_006399 | down | 2.7839003 | ALOX15 | NM_001140 | up | 2.4395845 |
| BDKRB2 | NM_000623 | down | 2.7283993 | ATP8B2 | NM_020452 | up | 3.2821248 |
| CA13 | NM_198584 | down | 2.313699 | BAG5 | NM_001015049 | up | 7.2899294 |
| CACNA2D2 | NM_001005505 | down | 3.7212276 | BF195775 | BF195775 | up | 2.5219588 |
| DKK1 | NM_012242 | down | 2.0091152 | BX091362 | BX091362 | up | 2.2093856 |
| DUSP1 | NM_004417 | down | 2.5269108 | C14orf144 | BC007749 | up | 2.0097556 |
| EGR2 | NM_000399 | down | 2.8463287 | C4orf26 | NM_178497 | up | 2.4465706 |
| ELOVL7 | NM_024930 | down | 2.3567688 | CA12 | AK000158 | up | 3.96613 |
| ENPP6 | NM_153343 | down | 2.1481051 | CIITA | NM_000246 | up | 2.4674168 |
| ERBB4 | NM_005235 | down | 2.5653942 | CR739597 | CR739597 | up | 2.6624396 |
| F5 | NM_000130 | down | 2.3298519 | CR748243 | CR748243 | up | 2.115664 |
| FAM101A | NM_181709 | down | 3.9106338 | CYP7A1 | NM_000780 | up | 2.0495083 |
| FAM101A | NM_181709 | down | 5.170043 | CYP7B1 | NM_004820 | up | 2.3924546 |
| FHOD3 | NM_025135 | down | 2.6699839 | EAF1 | NM_033083 | up | 2.0317638 |
| FLJ39822 | NM_173512 | down | 2.994576 | HLA-DQA1 | NM_002122 | up | 2.6678994 |
| FLJ44715 | AK126671 | down | 2.033678 | HLA-DQB1 | NM_002123 | up | 2.0056658 |
| FOSB | NM_006732 | down | 2.4567223 | HSF5 | NM_001080439 | up | 2.684714 |
| GATA1 | NM_002049 | down | 3.187345 | IRAK2 | NM_001570 | up | 3.8239567 |
| GRAP | NM_006613 | down | 2.54451 | IRX5 | NM_005853 | up | 2.2176828 |
| GUCA1B | NM_002098 | down | 2.851962 | LOC400756 | BC030752 | up | 2.082799 |
| HCP5 | L06175 | down | 2.1600773 | LOC643669 | XM_933621 | up | 2.1013808 |
| HORMAD2 | NM_152510 | down | 2.248619 | LOC652810 | XR_019510 | up | 2.0179825 |
| ICAM2 | NM_000873 | down | 2.0911252 | LRP4 | NM_002334 | up | 2.0070338 |
| IFI27 | NM_005532 | down | 2.4527955 | M27126 | M27126 | up | 2.053735 |
| IL17F | NM_052872 | down | 2.3185112 | MT1E | NM_175617 | up | 3.1026132 |
| IL1F7 | NM_014439 | down | 2.2045503 | MT1G | NM_005950 | up | 2.5606349 |
| KCNMB4 | NM_014505 | down | 2.3167608 | MT1H | NM_005951 | up | 3.1950183 |
| KIAA1505 | NM_020879 | down | 2.165876 | MT1L | X97261 | up | 2.8298597 |
| KIT | NM_000222 | down | 8.162423 | MT1X | NM_005952 | up | 2.9300673 |
| KLK11 | NM_144947 | down | 2.0223439 | MT1X | NM_005952 | up | 3.0408406 |
| LAMB3 | NM_001017402 | down | 2.0180652 | MT2A | ENST00000245185 | up | 3.6608098 |
| LENEP | NM_018655 | down | 2.9004846 | MT2A | NM_005953 | up | 3.5341341 |
| LTBP2 | NM_000428 | down | 2.633583 | MT2A | NM_005953 | up | 3.2365506 |
| MX1 | NM_002462 | down | 2.2560432 | NMUR2 | NM_020167 | up | 2.1342957 |
| MYO1D | NM_015194 | down | 2.1881073 | OBSL1 | AK023854 | up | 2.281759 |
| NPAL2 | NM_024759 | down | 2.160795 | RBM24 | NM_153020 | up | 2.3701508 |
| NR1H4 | NM_005123 | down | 2.1887279 | S73202 | S73202 | up | 2.1110113 |
| NUAK2 | NM_030952 | down | 2.8343716 | SCD5 | NM_001037582 | up | 2.1898105 |
| OSBPL6 | NM_032523 | down | 3.0237536 | SCN3A | NM_006922 | up | 2.409048 |
| OVOL2 | NM_021220 | down | 2.4823048 | SOST | NM_025237 | up | 3.1995454 |
| RBMY1A1 | NM_001007526 | down | 2.1988764 | SPNS2 | BC041772 | up | 2.152096 |
| RDH16 | NM_003708 | down | 6.2899146 | TBC1D10C | NM_198517 | up | 2.3046343 |
| RGS12 | NM_198229 | down | 2.061344 | TCP11L2 | NM_152772 | up | 2.2721546 |
| RTP4 | NM_022147 | down | 2.1016672 | TH1L | NM_198976 | up | 2.3462305 |
| SLC26A10 | NM_133489 | down | 2.936838 | THC2521656 | THC2521656 | up | 2.0013576 |
| SLC44A3 | NM_152369 | down | 2.0405505 | THC2648243 | THC2648243 | up | 2.5448472 |
| SPINK4 | NM_014471 | down | 2.329069 | THC2674191 | THC2674191 | up | 2.0647848 |
| SV2C | AB028977 | down | 2.1860335 | THC2729414 | THC2729414 | up | 2.6977122 |
| SYT13 | NM_020826 | down | 3.344947 | TPPP2 | NM_173846 | up | 2.2527223 |
| TDGF1 | NM_003212 | down | 5.396204 | TRIM17 | NM_016102 | up | 3.9586205 |
| TGFA | NM_003236 | down | 2.1505268 | WNT11 | NM_004626 | up | 2.210344 |
| TXNRD2 | NM_006440 | down | 2.789754 |  |  |  |  |
