# Supplementary material for: GATA6 Activates Wnt Signaling in Pancreatic Cancer by Negatively Regulating the Wnt Antagonist Dickkopf-1
Source: PLoS One. 2011 Jul 19;6(7):e22129. doi: 10.1371/journal.pone.0022129 (PMC3139620; doi:10.1371/journal.pone.0022129)
Supplement: File S1 — Oligonucleotide Sequences Used in Current Study. (DOC) [file pone.0022129.s007.doc]

**File S1: Oligonucleotide sequences**

RT-qPCR Primer sequences

| **Gene** | **Accession Number** | **Forward** | **Reverse** | **Product Size** |
| --- | --- | --- | --- | --- |
| CTNNB1 | NM_001098209 | gttctcctcagatggtgtctg | ggtgaacaaagcattttcacc | 170 |
| DKK1 | NM_012242 | gccccgggaattactgcaaaaatg | ccggagacaaacagaaccttcttgtc | 210 |
| DKK2 | NM_014421 | gatcgaaaccacggtcattac | gcactggtttgcagattttgg | 178 |
| DKK3 | NM_001018057 | ctgtgtgtctggggtcactg | gctctagctcccaggtgatg | 206 |
| DKK4 | NM_014420 | gggacaagagggagaaagttg | ctggaagatttctggagcttg | 163 |

**EMSA Probe sequences**

GATA-#1: 5’-ACGCGTCTGCCTAATCAAGTTCATCTA-3’

mGATA-#1: 5’-ACGCGTCTGCCTccTCAAGTTCATCTA-3’

GATA-#2: 5’-ATCTACCGCCGCGATTGCCCTGATT-3’

mGATA-#2: 5’-ATCTACCGCCGCGAggGCCCTGATT-3’

GATA-#3: 5’-AAATGGTTTGATTATCGGATGGT-3’

mGATA-#3: 5’-AAATGGTTTGAggATCGGATGGT-3’

GATA-#4: 5’-TTGCAGAGCCTATCACCCCTCGGCTC-3’

mGATA-#4: 5’-TTGCAGAGCCgAgCACCCCTCGGCTC-3’

Positive control derived from *TFF2* promoter containing a GATA6 binding site: 5’-GCCAGCAGATAGCATGGAAAAG-3’ (Al-azzeh ED, Fegert P, Blin N, Gott P. Transcription factor GATA-6 activates expression of gastroprotective trefoil genes TFF1 and TFF2. Biochim Biophys Acta 2000;1490(3):324-32).

**CHIP primer sequences (see also Supplemental Figure 3)**

GATA binding motifs #1-3 (size, 160 bp)

5’-CTGTCCGCTTTGGTCC-3’ (forward)

5’-CTGGCGTTTAGAAATTAGATC-3’ (reverse)

GATA binding motif #4 (110 bp)

5’-CAGCGAGTATTGGCATAA-3’ (forward)

5’-CCTGGCAGTAAGGGTTG-3’ (reverse)
